# The Mobilizing Action Toward Community Health Partnership Study: Multisector Partnerships in US Counties with Improving Health Metrics

**DOI:** 10.5888/pcd11.130103

**Published:** 2014-01-09

**Authors:** Susan J. Zahner, Thomas R. Oliver, Kirstin Q. Siemering

**Affiliations:** Author Affiliations: Thomas R. Oliver, Kirstin Q. Siemering, University of Wisconsin–Madison, Madison, Wisconsin.

## Abstract

**Introduction:**

Multisector partnerships are promoted as a mechanism to improve population health. This study explored the types and salient features of multisector partnerships in US counties with improving population health metrics.

**Methods:**

We used the “Framework for Understanding Cross-Sector Collaborations” proposed by Bryson, Crosby, and Stone to guide data collection and interpretation. Comparative case studies were conducted in 4 counties selected on the basis of population, geographic region, an age-adjusted mortality decline better than the US average, and stable per capita income. Data were collected through website and report reviews and through in-depth interviews with key informants (N = 59) representing multiple sectors. County reports were developed and cross-case themes related to partnership types and salient features were derived.

**Results:**

Multisector collaboration was common in all 4 counties despite substantial variations in population, geographic size, demographic diversity, and other characteristics. Most partnerships were formed by professionals and organizations to improve delivery of health and social services to vulnerable populations or to generate policy, system, and environment changes. Multisector collaboration was valued in all cases. Outcomes attributed to partnerships included short- and long-term effects that contributed to improved population health.

**Conclusion:**

The Bryson, Crosby, and Stone model is a useful framework for conducting case study research on multisector partnerships. Outcomes attributed to the multisector partnerships have the potential to contribute to improvement in population health. Further study is needed to confirm whether multisector partnerships are necessary for improving population health within counties and to understand which partnership characteristics are critical for success.

## Introduction

One method for improving the performance of a complex system of policies and services is to reassign responsibility and power within the system (1). Because determinants of health often lie outside the formal jurisdiction of public health agencies, improving population health and community well-being may depend on securing authority, resources, and commitment from leaders in many county sectors. Cross-sector collaboration is increasingly viewed as necessary to improving community health (2). Multisector partnerships to improve population health typically involve collaboration among the government, nonprofit, and business sectors of a geographic area and often include leaders, staff members, and resources from the fields of education, economic development, housing, transportation, agriculture, health care, public safety, community services, and public health organizations (3).

Research has identified partnership qualities and contextual conditions that support effective multisector collaboration, including having sufficient resources, a common vision, strong leadership, clear structures and processes, a broad array of partners, prior ties, and relationships marked by mutual trust, respect, and commitment to the cause (2–4). However, evidence of the effectiveness of multisector partnerships in improving population health outcomes is limited (3). Increased understanding is needed of the characteristics of those multisector partnerships that are associated with population health improvement and of whether such partnerships are necessary to improve population health (5). To help fill these research gaps, we conducted the Mobilizing Action Toward Community Health (MATCH) partnership study, which we describe in this article. 

If multisector partnerships are necessary for improving population health, then we would expect to find such partnerships in places with improving population health outcomes. If multisector partnerships exist in places with such outcomes, then the characteristics of those partnerships could provide useful information for researchers and practitioners planning to use partnership strategies to improve population health. Our objective was to explore the types and characteristics of multisector partnerships in defined geographic areas in which population health metrics have substantially improved during the past 30 years.

## Methods

We used a comparative case study design appropriate for understanding phenomena that are complex and context-sensitive (6). Study methods were reviewed and approved as exempt research by the University of Wisconsin–Madison Social Sciences internal review board.

We used the county as our unit of analysis because data relevant for identifying improvement in population health are available by county, and counties are typical jurisdictional boundaries for public health initiatives. Four case counties were chosen from among all US counties by using an iterative process with mortality data drawn from CDC Wonder (http://wonder.cdc.gov/mortSQL.html) and population demographics and income data from the US Census for 1982 through 2006 (http://www.census.gov/). First, we searched for counties that demonstrated better-than-US-average declines (>28.1%) in age-adjusted mortality over a 20-year period (1982–1986 compared with 2002–2006) and a better-than-US-average decline (>6.7%) in age-adjusted mortality rates from 1997–2001 to 2002–2006. We excluded counties that may have achieved mortality declines because of rapid changes in demographics or wealth by applying a second criterion, the change in per capita income from 1990 to 1999 within 5% or below the US average percentage change in income over that period (0.58%). Application of these criteria yielded an initial list of 71 counties. Contiguous counties within 2 major metropolitan areas (New York City and Washington, DC) were excluded to allow a focus on the experience of single counties. We next gave priority to improvement in the more recent period by using the criterion of approximately twice the average decline (>13%) in mortality from 1997–2001 to 2002–2006. These steps resulted in a list of 35 counties. We then considered population size, percentage of African Americans, median household income, and geographic region (based on Federal Emergency Management Agency regions and only 1 per state) to achieve variation in county characteristics. One of the initially selected counties declined to participate and was replaced with another county on the list from the same state.

We invited counties to participate through a telephone call to the director of the county health department. The health department directors were asked to select or suggest people for us to interview: key members of multisector collaborations in the county, including staff members of the health department itself, public schools, county policy makers, law enforcement, social services agencies, health care facilities, chambers of commerce (business sector), and community organizations. In total, we received the names of 94 potential interviewees, who were all invited to participate in our study through electronic letters of invitation followed by telephone calls to schedule interviews.

The Framework for Understanding Cross-Sector Collaborations (4) (Figure) proposed by Bryson, Crosby, and Stone guided data collection and interpretation. The framework provides a model for understanding cross-sector collaboration that encompasses contextual factors and antecedents leading to partnership formation, collaborative processes, structural characteristics, constraints, and contingencies that affect partnership functioning and partnership outcomes. We created semi-structured interview guides based on the framework (Appendix).

**Figure:**
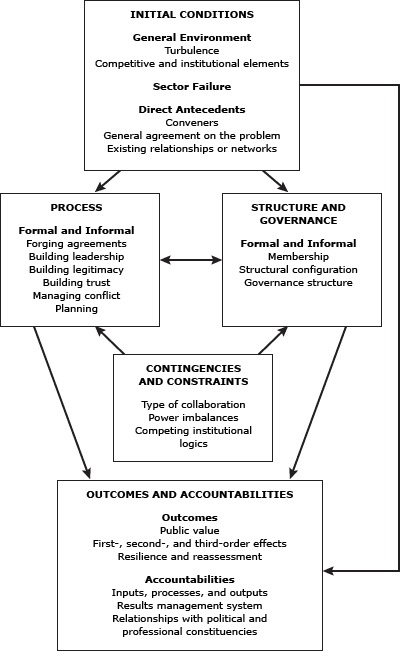
A Framework for Understanding Cross-Sector Collaboration (4). Reprinted with permission from Bryson JM, Crosby BC, Stone MM. The design and implementation of cross-sector collaborations: propositions from the literature. Pub Admin Rev 2006; Special Issue: p. 45.

From September through December 2010, 2 researchers conducted in-person or telephone interviews with those who agreed to participate in our study. The interviews were audio-recorded and professionally transcribed (3 exceptions were due to interviewee preference or equipment failure). Websites and relevant documents, such as county health assessments, action plans, annual reports, and US Census data were used to develop county profiles to support analysis of each case study.

We used deductive content analysis methods to derive partnership types and salient features from the data (7). Partnerships were classified by membership and by organizational structure by using a typology developed by Butterfoss (8). In this typology, coalitions are defined by membership and structure. Grassroots coalitions form in times of crisis to pressure policy makers. Agency-based coalitions are formed by professions in times of crisis or as a long-term approach to increase influence. Community-based coalitions include both professional and grassroots members and are formed to influence long-term health and welfare practices in communities. Organization-set coalitions are made up of cooperative organizations that provide resources or services under an umbrella organization. Network coalitions are made up of organizations that each provides services only to a particular population. Action-set coalitions are formed to deal with a specific issue and may be informal or formal (8). We classified characteristics by using a coding scheme developed from the Bryson, Crosby, and Stone framework (4) before beginning the coding process. One researcher read and coded the transcripts of each interview, adding new codes within the major categories of the basic coding scheme as warranted by the data. Coding was verified by a second researcher. Reports were written for each county, and cross-county themes were derived.

## Results

Studies were conducted in Chittenden County, Vermont; Kane County, Illinois; Fulton County, Georgia; and Orange County, California. A total of 59 county coalition members were interviewed (62.7% of those invited to participate) (Table 1).

**Table 1:** Interviewees by County and by Sector, Four US Counties With Improving Health Metrics, September–December, 2010

County	N	Public health^a^	Health Care	Social services^b^	Business	Education (Kindergarten-12)	Funder	Community-Based Organizations^c^	Government^d^
Kane	13	3	4	--	--	1	--	4	1
Chittenden	16	2	1	1		1	2	5	4
Fulton	15	2	4	2	2	2		2	1
Orange	15	6	3	2	--	--	2	2	--
Total	59	13	12	5	2	4	4	13	6

Abbreviation: __, not applicable.

a Includes state and local public health officials.

b Includes local and county governmental social services.

c Includes community organizations (faith-based and other non-profit organizations).

d Includes elected officials and government agency staff not included elsewhere.

We found many multisector partnerships in all 4 counties (Table 2). Most were organized either as community-based coalitions or as organization-set coalitions and were created by professionals and organizations to improve access to and coordination of health and social welfare services to vulnerable individuals and families. Some were formed in response to a particular need found through community assessment processes. The range of partnership goals described by interviewees included the following:

**Table 2:** Multisector Partnerships in Four US Counties with Improving Health Metrics, September –December, 2010

Case	Types of Partnerships
Kane County, Illinois	***Membership***
	**Community-based coalitions**
	• Making Kane Fit for Kids
	• Circle of Wise Women
	• Kane Community Health Access Integrated Network (KCHAIN)
	***Organizational structure***
	**Organization-set coalition**
	• Aurora Primary Care Consortium (also a nonprofit organization)
	• Kane County Perinatal Committee
	• Campañeros En Salud (also a nonprofit organization)
	**Network coalition**
	• Kane County Mental Health Council
	• All Our Kids Network (AOK)

Chittenden County, Vermont	***Membership***
	**Community-based coalitions**
	• Champlain Initiative
	• Regional Hunger Councils (affiliated with Hunger Free Vermont, a nonprofit organization)
	• Activate Vermont
	• Safe Streets Collaborative
	***Organizational structure***
	**Organization-set coalition**
	• KidSafe Collaborative of Chittenden County (also a nonprofit organization)
	• Building Bright Futures (regional councils)
	• Support and Services at Home

Fulton County, Georgia	***Membership***
	**Community-based coalition**
	• Atlanta Beltline
	***Organizational structure***
	**Organization-set coalition**
	• Common Ground Initiative
	• Westside Wellness Zone
	**Action-set coalition**
	• Drake House
	• Community Improvement District
	**Network coalition**
	• Fulton Family Care Network
	• Project CEASE

Orange County, California	***Membership***
	**Community-based coalition**
	• Nutrition and Physical Activity Collaborative
	***Organizational structure***
	**Organization-set coalition**
	• Health Funders Partnership of Orange County
	• Orange County Children’s Partnership
	• FaCT: Families and Communities Together
	• Coalition of Orange County Community Health Centers

- Improving access to health care (primary, specialty, perinatal, and mental health)
- Improving coordination of health and social services
- Creating policy, system, and environment changes to promote health, particularly in the areas of nutrition, physical activity, safety, and perinatal health
- Creating early childhood education and health initiatives to ensure children are ready to learn

### General environment

The 4 counties varied by geographic region, population size, ethnic/racial diversity, income, and political environment. We found similarities among the counties in social, organizational, and policy contexts for multisector partnerships. Most interviewees reported their county to be a desirable place to live, with recreational and physical environment characteristics that supported healthy lifestyles. Traditions of community engagement, volunteerism, and social cohesion featured strongly in interviews from 2 counties. Multisector collaboration and coordination of services were reported as common in 3 of the counties. As one interviewee stated, “Routinely, comfortably . . . we plan together, and design programs together . . . there is a tremendous amount of trust that yes, we’re in this together and we can do this.”

Competition for resources was reported as problematic in 1 county. However, interviewees from that county reported increasingly turning to collaborative models for addressing health and social needs of county residents. Health care and social services systems were generally considered strong or improving in all 4 counties. Public health systems were organized differently: independent local health departments in 3 counties and combined health and human services agencies in 1 county; all were reported as strong and increasingly focused on addressing health disparities.

Collaboration was common in all 4 counties. A sense of community, a tradition of community engagement and volunteerism, and a strong health care, social service, and public health service delivery system were reported as supporting multisector collaboration.

### Factors leading to partnership initiation

In all 4 counties, interviewees reported partnerships that were initiated in response to collaborative community-needs assessment processes of various types. For example, an interviewee stated, “Doing that study 5 years ago now gives us baseline data on which we can overlay everything since then. . . . All that gives us an ability to have a voice about the projects in a different way.”

In 1 county, the local needs assessment process was state-mandated, and in 2 counties the needs assessment process emerged from voluntary nonprofit organizations. Leadership was a second driver in all counties. Interviewees described key leaders as intentional, strong, visionary, and charismatic. Leadership was reported as coming from public health, health care, government, and business sectors and from individuals who champion specific causes or who are social entrepreneurs. Additional drivers were having trusted relationships and collaborative experiences with coalition members on other issues.

In at least 2 of the counties, we identified the following 3 drivers: 1) an organization serving as a neutral convener, 2) changes in community benefits regulations resulting in more hospitals conducting community assessments and engaging in partnerships to address identified needs, and 3) a funder that expected or required collaboration among organizations within the county. In addition, in 2 counties, we found a connection between the existence of partnerships and a wide recognition and acceptance of the multiple determinants of health. Finally, we found that economic adversity and tight budgets contributed to the need to collaborate across sectors. Our cross-county analysis of common antecedents and drivers of multisector collaboration showed 3 main themes: 1) formally identified community needs frequently drive multisector partnerships on health issues, 2) leadership is a powerful driver in multisector partnerships and may come from multiple sectors, and 3) prior relationships promote continued collaboration.

### Processes

Interviewees in all 4 counties reported that planning processes such as visioning, identifying a common mission, and articulating clear goals supported multisector collaboration. Good facilitation and management processes such as formal agreements, voting procedures, documentation of partnership actions, and regular meetings to support partnership activities were reported in 3 of the counties. One interviewee’s comment illustrates the importance of these processes: “I’m a juggler, I’m a bus driver, and I herd the cats . . . unless you have a strong manager fulfilling those roles, the projects don’t move forward.”

Staying focused on action and being successful in accomplishing visible positive results were commonly reported. Engaging the right people, including leaders of key organizations, was frequently noted as a strategy for successful partnership processes. The participation, active leadership, and in some cases substantial financial support from major health care provider organizations were important in all counties. Having resources, including access to students and in-kind organizational support, was viewed as important to sustaining partnership structures and processes in all 4 counties.

### Structure

We found a variety of partnership structural configurations and learned that partnership structures evolved over time. For example, some partnerships were described as beginning as small, focused efforts initiated through community assessment processes or grant projects and evolving to become nonprofit organizations or structures sustained through policy changes. Common organizational structures included steering or advisory committees or commissions with cross-sector representation from relevant partners and policy-makers. Having a formal agreement (ie, a memorandum of understanding) was a structure mentioned in 2 counties, and a consortium of funders that pool resources for projects was a partnership structure in 2 counties.

### Constraints

Interviewees spoke about factors that constrain or hinder formation of cross-sector partnership, functioning, and outcomes. Although resource limitations at times spurred community organizations to form partnerships, lack of resources was also a factor that hindered further development of established partnerships and kept them from reaching their full potential. As one interviewee explained, “Things that hinder are the scarcity mentality that so many of us have . . . and so they’re hesitant to get involved in more things. Many organizations have less staff than they did 5 years ago so there are fewer bodies to . . . engage in this type of activity.”

Competition for resources (turf issues) and challenges associated with the process of collaboration were noted as constraints by interviewees in 2 counties. Staff turnover, lack of leadership, and poor facilitation were also hindrances to partnership in 2 counties. In addition, interviewees in 2 counties noted that politics hindered partnership: for example, when some members or organizations had more power than others, they tended to dictate the direction and pace of partnership activities.

### Outcomes

In all cases, interviewees reported that outcomes of multisector partnerships improved access to services through 1) better coordination across and within health and social services organizations, 2) more comprehensive approaches to meeting community needs, and 3) leveraging new resources and creating new services through partnerships. Another type of outcome described in all 4 counties reflected improvements in child health, nutrition, and physical activity. These outcomes were associated with policy changes and increased emphasis on health promotion, nutrition, and safety initiatives in communities, often in response to community needs assessments. Improvements in built environments such as traffic calming, pedestrian walkways, lighting, and community gardens were noted as partnership outcomes in 3 counties. In summary, we found that multisector partnerships were reported to achieve short-term outcomes and create public value by better coordinating the services and assets of partner organizations, offering comprehensive approaches, and focusing on policy, system, and environment changes that contribute to improvements in population health.

## Discussion

Most studies of cross-sector collaboration focus on efforts related to specific program areas or types of partner organizations. This study advances the science on collaboration as a public health strategy by providing information about the types and salient features of multisector partnerships in counties with demonstrated improvements in population health metrics. Population size, geographic region, and median wealth do not appear to have a strong connection to the formation and operation of multisector partnerships relevant to improving population health. Multisector partnerships existed everywhere we looked and varied in origin, purpose, and activities. The types of partnerships identified most frequently focused on improving access to and coordination of health care and social services and on supporting child health and safety. However, we also found examples of collaborating on upstream determinants of health and activities designed to address chronic disease by using the paradigm of “health in all policies,” which focuses on changing policies, systems, and environments to ensure sustainable improvements in conditions that support health promotion and disease prevention.

We found the Bryson, Crosby, and Stone framework (4) useful for guiding data collection and interpretation. Frequent use of this framework could help standardize measurement strategies in multisector collaboration research. Many partnership features identified in the literature on collaboration were supported in this study (2–4). Leadership, relationship building, prior collaborative ties, assessments to drive planning and action, common goals and vision, skilled facilitation, and sufficient resources were key drivers and supports for partnership activity. Engaging stakeholders in the partnership, having the right people from relevant institutions and communities as well as key leaders at the table were important processes for all 4 counties. Partnerships evolve in form and activity to accommodate newly identified needs and new partners. Multisector partnership connections are used to tap a variety of sources for expertise and technical assistance needed to pursue partnership goals and activities. Resource limitations both motivate partnerships and constrain them from being effective.

Our findings confirmed prior expectations for finding robust multisector partnerships in places characterized by improving population health metrics. Most interviewees extolled the value and the absolute necessity of multisector collaboration in their communities. The outcomes attributed to such partnerships created public value and included positive short- and long-term effects that logically are precursors to improvement in population health.

Limitations inherent in the study design and methods are relevant to consider when interpreting our results. Our county selection process was driven by only 1 measure of population health improvement. Improvement could be defined by many metrics, not just better-than-average declining mortality rates. The number of counties was limited by resources and was not fully representative of all counties with improving health metrics. The selection of key interviewees for each county depended on the local health department director. This recruitment process resulted in a sample of knowledgeable interviewees across relevant sectors but could have resulted in a biased sample if the director avoided selecting or suggesting individuals who were not supportive of partnerships. In addition, interviewees from other relevant sectors, such as housing, were missing from the data. An interview guide was used as a reference during the interviews but not strictly followed because of the semistructured interview format. As a result, information addressing every aspect of the Bryson, Crosby, and Stone framework was not elicited from every interviewee.

This study cannot explain in a causal way the improving population health metrics in the 4 counties studied. Data were not collected through similar studies from counties with health metrics that show worsening outcomes. Therefore, we were not able to infer whether the findings in these counties differ from counties of similar size and location but with worsening population health metrics. Longitudinal, comparative studies are still needed to determine whether multisector partnerships are necessary for achieving improvements in population health in geographic areas and to understand what partnership characteristics are most critical for ensuring success.

## Appendix. Interview Guide^a^

1. We chose to include [NAME COUNTY] in our study because of your sustained record of better-than-average declines in age-adjusted mortality rates while having average or below average change in per capita income over the same time period. Population health by this broad measure seems to be improving in this county.
  - What are your immediate thoughts about factors that are contributing to population health improvement in this county?
  - How would you describe the overall population health status of people in this county?
2. We would like to understand more about the general social, organizational, and policy environment of this county.
  - How would you describe the overall social, organizational, and policy environment?
  - How would you describe the rate of change in this county?
  - How would you describe the network or system of social and health organizations with regard to complexity and cooperativeness?
  - How active or engaged are formal policy-makers in this county?
3. What policies, programs, practices, and partnerships do you think might be contributing in important ways to population health improvement in this county?
4. We would like to understand more about interorganizational partnerships in [NAME COUNTY]. Is partnership a common approach to addressing health or social problems in this county?
  - What types of interorganizational partnerships or collaborations exist in this county?
  - We define a multisector partnership as a collaboration that crosses organizational sectors within a geographic area and may include government agencies, businesses, nonprofit organizations, schools, communities, and the public. Are there any multisector partnerships aimed specifically at improving health or social conditions in this county?
5. Consider one multisector partnership in which your organization is now, or has been, engaged and which you think is contributing to or has contributed to population health improvement in this county.
  - What are the main goals of the partnership?
  - How did this partnership start?
6. We are interested in the structure of multisector partnerships.
  - What organizations are involved?
  - How is membership in the partnership structured?
  - How is the partnership governed?
7. We are interested in the resources available to the partnerships. How are they funded?
8. We are also interested in the processes of these multisector partnerships.
  - Are there formal agreements that guide the work of the partnership?
  - How does leadership of the partnership work?
  - How is the partnership funded or supported?
  - Has the partnership developed a plan that guides its activities?
  - How does the partnership manage issues such as trust and conflict?
9. We are interested in your perspective on issues that support or hinder the success of multisector partnerships.
  - What factors do you think support success for this partnership?
  - What factors do you think limit success for this partnership?
10. What outcomes do you attribute to this partnership?
11. How important are these multisector partnerships to improving health or social conditions in this county?
12. Is there anything else you would like to tell us about your county’s efforts to improve the health of its people?

^a^ This questionnaire was used as a semistructured interview guide; not all questions were asked of every interviewee, and questions asked were not always phrased exactly as they are written in the guide.
